# Modeling RNA duplex dynamics with Gibbs sampling enhances base-pair prediction accuracy and reveals structural activity profiles

**DOI:** 10.1093/nargab/lqaf099

**Published:** 2025-07-17

**Authors:** Simon Chasles, François Major

**Affiliations:** Department of Computer Science and Operations Research, and Institute for Research in Immunology and Cancer, Université de Montréal, Montréal, Québec H3C 3J7, Canada; Department of Computer Science and Operations Research, and Institute for Research in Immunology and Cancer, Université de Montréal, Montréal, Québec H3C 3J7, Canada

## Abstract

The RNA secondary (2D) structure prediction problem consists in determining the set of base pairs that form within an RNA molecule from its sequence. A related task is the RNA hybridization problem, where two RNA strands interact to form a duplex. Thermodynamics-based methods typically rely on experimentally determined energy parameters to compute minimum free energy structures for both single-stranded RNAs and duplexes. Through the Boltzmann distribution, these parameters can be used to estimate base-pairing probabilities. Here, we leverage these probabilities to simulate RNA:RNA interaction dynamics. Inspired by the Ising model, we apply Gibbs sampling to model the stochastic formation and disruption of base pairs over time in RNA duplexes, ultimately deriving a consensus structure. The resulting method, MC-DuplexFold (mcdf), enhances base-pair prediction accuracy when integrated with other RNA 2D structure prediction algorithms. Through benchmarking, we reaffirm the previously observed trend that approximate or heuristic methods, such as RIsearch and Sfold, outperform exact methods like RNAcofold and DuplexFold in structural prediction accuracy. Additionally, mcdf provides structural activity statistics that can be incorporated into the modeling of miRNA primary transcripts, precursors, and target interactions, thereby refining predictions of miRNA:mRNA duplex dynamics.

## Introduction

RNA folding is an increasingly important area of research, driven by its central role in many biological processes, from gene regulation to protein synthesis. Despite significant progress, current methods for predicting RNA structures, including duplex formation, remain limited, often failing to account for the complexity of RNA’s dynamic nature and the influence of cellular conditions [1]. Accurately predicting RNA duplex structures, where two RNA strands form base-pairing interactions, has vast applications in areas such as drug design, RNA-based therapeutics, and understanding gene regulatory networks [2, 3].

As a prominent component of post-transcriptional gene regulation, RNA interference (RNAi) [4, 5] constitutes a particularly interesting field of study for computational biology due to its relevance in cancer development [6] and the complexity of its underlying interaction networks [7, 8]. In humans, over 2000 microRNAs (miRNA) [9] modulate the expression of nearly all messenger RNA (mRNA) transcripts [10, 11] which calls for important computations. Yet, accurate modeling of these networks requires a thorough understanding of miRNA:mRNA interactions and RNA:RNA interactions in general.

Mature miRNAs are composed of 20–26 nucleotides [12]. They are loaded in Argonaute proteins to form the RNA-induced silencing complex (RISC) [13]. Guided by the miRNA, the RISC can bind to single stranded RNA, and in the case where it binds to mRNA, translational repression can occur [14]. Considerable effort has been invested in determining which miRNA–mRNA couples can hybridize, meaning they can form a stable RNA duplex structure. As a general rule, sequence complementarity through Watson–Crick base pairs in the seed region mediates most of target recognition [15], but it is expected that the structure of the whole duplex along with its structural dynamics and defects like bulges, mismatches, and noncanonical base pairs can better explain the whole phenomenon [16, 17].

Understanding the structural dynamics of RNA duplexes is not only related to mRNA targeting by miRNAs, but also to miRNA maturation pathways [18]. When being processed by Drosha and Dicer, the precursor miRNA adopts a hairpin structure of ≈30 base pairs which acts as a dynamic RNA duplex [19]. Single nucleotide polymorphisms (SNPs) can impact the maturation efficiency of miRNAs [20] and the mechanisms governing this phenomenon have yet to be understood [21]. As sequence and structure do not suffice to explain the whole maturation process, the integration of kinematic information can prove useful [22].

We are thus motivated by implementing a method that can characterize known RNA:RNA interactions from sequence, both in terms of base-pair prediction and 2D structure dynamics. On the one hand, experimental methods struggle to determine miRNA:mRNA duplex structures in the presence of the RISC. On the other, most computational methods solely predict a single structure consisting of GC, AU, and GU base pairs, although various algorithms incorporate noncanonical base pairs as they significantly impact general structure formation [23–28]. Even if conformational space exploration allows for RNA motif probability computation [29, 30], the dynamic nature of miRNA:mRNA interactions makes the evolution of 2D structures through time a key element to take into account when modeling RNA duplex structures [31, 32].

To address these concerns and to improve prediction accuracy in general, we propose MC-DuplexFold (mcdf), a Gibbs sampling algorithm that simulates the formation and disruption of base pairs in RNA:RNA interactions [33]. The algorithm visits the most probable conformations of an RNA duplex before returning a consensus structure with noncanonical base pairs. In comparison with methods that compute suboptimal structures (in terms of energy minimization) along with base-pairing probabilities [25, 34–36], mcdf provides frequencies of occurrence for the most stable structures together with structural activity statistics like transition frequencies and entropy estimations. It should be noted that mcdf is not primarily intended for predicting the location of RNA:RNA binding sites, but rather for studying the structural dynamics of known interactions.

Here, we illustrate that mcdf can identify unstable base pairs in miRNA:mRNA duplexes and predict biologically relevant features like miRNA maturation efficiency and miRNA:mRNA dissociation constants (*K*_d_). Also, by extracting helices from complete RNA structures determined by nuclear magnetic resonance (NMR) or X-ray crystallography [37], we quantify the performances of mcdf and other 2D structure prediction algorithms on a base-pair detection task. We observe that mcdf presents enhanced base-pair prediction accuracy when used in conjunction with other algorithms. Furthermore, we notice heuristic or simplified free-energy minimization methods like RIsearch and Sfold perform better than exact methods like RNAcofold or DuplexFold, suggesting inaccuracies in the thermodynamic model render exact optimization unnecessary.

## Materials and methods

### The RNA hybridization problem

Let *s* ∈ Σ^*n*^ (from 5′ to 3′) and *t* ∈ Σ^*m*^ (from 3′ to 5′) be two RNA sequences, where $\Sigma = \lbrace \texttt {A}, \texttt {C}, \texttt {G}, \texttt {U}\rbrace$ is the RNA alphabet. Assume *s* and *t* interact such that *T* = {(*i*, *j*) | *s*_*i*_and *t*_*j*_ form a base pair} ⊆ {1, …, *n*} × {1, …, *m*} is the set of ground truth base pairs determined experimentally [38, 39] with annotations [40, 41]. The task is to predict *T* from *s* and *t*. We assume nucleotides can only pair once so ∀{(*i*, *j*), (*u*, $v$)} ⊆ *T*: *i*≠ *u*∧ *j*≠ $v$. We also assume base pairs cannot cross so ∀{(*i*, *j*), (*u*, $v$)} ⊆ *T*: *i* < *u*↔ *j* < $v$. As detailed in the supplementary data, the number of possible structures is, under these assumptions, given by $\binom{n+m}{n} \sim \frac{1}{\sqrt{\pi n}}4^n$ when *m* ∼ *n*. We denote the set of possible structures by $\mathcal {S}$.

As the number of possible structures grows exponentially with the number of nucleotides, the predicted structure must be selected in an efficient manner. The maximum likelihood approach is to output the minimum free energy (MFE) structure using dynamic programming by considering the sum of energy contributions of RNA loop motifs formed by canonical base pairs, stacks, bulges, and interior loops. In general, such an approach yields high precision but low recall as noncanonical base pairs are ignored. To predict noncanonical base pairs, one can use, for instance, MC-Fold [25], an MFE algorithm that uses statistics on base-pair frequencies in the PDB to estimate energy contributions for noncanonical base pairs. Such a method, on the other hand, yields high recall but low precision, as it tends to overestimate the contributions from noncanonical base pairs. We are thus motivated by proposing a method to balance the precision-recall tradeoff while providing structural activity statistics about the base pairs in the duplex.

### Mathematical model

We draw our inspiration from the Ising model to define a base-pair formation model [42]. We define a probabilistic graphical model with *nm* variables to represent the set of possible base pairs. Each variable *X*_*ij*_ takes value 1 if *s*_*i*_ and *t*_*j*_ form a base pair and 0 otherwise. Just like in the Ising model, there exist dependencies between neighboring variables, but here, we allow these dependencies to evolve through time with respect to the state *X* of the model. Namely, we consider two variables *X*_*ij*_ and *X*_*uv*_ (with *i* < *u*) to be neighbors if there is no base pair between them in the current state and if the gap (*u* − *i*, $v$ − *j*) allows MC-Fold [25] to estimate the energy contribution of the nucleotide cyclic motif formed by these two consecutive base pairs. More precisely, *X*_*ij*_ and *X*_*uv*_ are considered neighbors if (*u* − *i*, $v$ − *j*) ∈ {1, 2, 3, 4}^2^∖{(3, 4), (4, 3), (4, 4)} and if ∀(*a*, *b*) such that *i* < *a* < *u* and *j* < *b* < $v$, *X*_*ab*_ = 0. We denote the set of neighbors of *X*_*ij*_ as *N*_*ij*_.

From the factorization of the distribution given by the probabilistic graphical model described in the last paragraph, there exist real numbers η_*ij*_ and η_*ij*; *uv*_ such that we can express the likelihood of the system to be in state *X* = *x* as the following, where *Z* denotes the partition function [43].


\begin{eqnarray*}
p(x) = \frac{1}{Z}\exp {\left\lbrace \sum _{\text{pairs } ij}\eta _{ij}x_{ij} + \sum _{\text{neighbors }ij;uv}\eta _{ij;uv}x_{ij}x_{uv} \right\rbrace }
\end{eqnarray*}

To apply Gibbs sampling, once a base pair (*i*, *j*) is selected, we compute its pairing probability $\mathbb {P}\left[X_{ij} = 1 | X_{-ij} = x_{-ij}\right]$ given the state *x*_−*ij*_ of all other variables in the model [43]. We use the following properties to compute this probability, where $\sigma (z) = \frac{1}{1 + \exp \lbrace -z\rbrace }$ denotes the sigmoid function.


\begin{eqnarray*}
\mathbb {P}\left[X_{ij} = 1 | X_{-ij} = x_{-ij}\right] &=& \sigma \left(\eta _{ij} + \sum _{x_{uv} \in N_{ij}} \eta _{ij;uv}x_{uv}\right)\\ &\approx & \sigma \left(\frac{-\varepsilon (i, j, N_{ij}, x_{-ij})}{RT}\right)
\end{eqnarray*}

The main trick is to approximate this probability using MC-Fold’s estimation ϵ(*i*, *j*, *N*_*ij*_, *x*_−*ij*_) of the energy contribution of the formation of base pair (*i*, *j*) given the state *x*_−*ij*_ of its neighbors *N*_*ij*_. Intuitively, the η_*ij*_ parameters capture the stability of base pairing interactions while the η_*ij*; *uv*_ parameters capture the stability of base stacking interactions, which are both simultaneously taken into account by ϵ(·). By establishing a parallel with the Boltzmann distribution, we introduce the hyperparameter *RT* to scale the influence of ϵ(·) on the pairing probabilities as depicted in Supplementary Fig. S1. As *RT* grows, the randomness of the system increases, which provides us with the means to figuratively control the temperature of the simulation.

We must keep in mind that while the use of ϵ parameters prevents us from estimating η parameters, this does not mean our model is free from statistical learning. The ϵ parameters were estimated using statistical inference over 531 PDB structures [25] which must be considered as seen by mcdf when measuring performances. At the same time, this provides us with a training set to tune the hyperparameters of the model like the temperature parameter *RT* and others described in next section.

### Gibbs sampling and structure prediction

We execute a Markov chain Monte Carlo (MCMC) simulation by applying Gibbs sampling. Since we are using MC-Fold’s parameters, the initiation state of the system is the MFE structure as computed by MC-FlashFold [44], a fast variant of MC-Fold that computes pseudoknot-free structures in cubic time through dynamic programming. At each simulation step, we randomly select one pair (*i*, *j*) uniformly within a list of candidates composed of the base pairs in the actual state and their neighbors (the empty structure is not allowed). Once a pair (*i*, *j*) is selected, we compute its pairing probability *p* as described in last section, sample a number $\xi \sim \mathcal {U}(0, 1)$ and set *X*_*ij*_ to 1 if ξ < *p* and 0 otherwise. We thus form and break base pairs one at a time to explore the conformational space of an RNA duplex.

**Figure 1.**
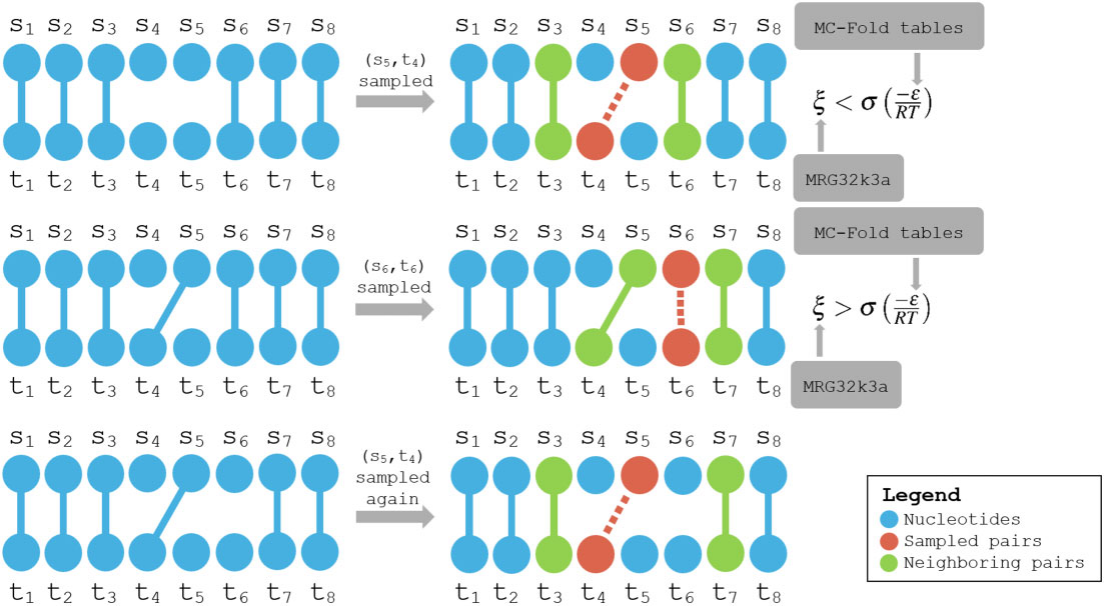
Functioning of the mcdf algorithm. *Top:* Given a duplex structure, a pair of nucleotides is randomly selected from the set of existing base pairs and their neighbors, as defined in the text, namely, those within a short cyclic motif gap and not separated by intervening base pairs. In this example, the candidate pair (*s*_5_, *t*_4_) is considered a neighbor of the existing base pairs (*s*_3_, *t*_3_) and (*s*_6_, *t*_6_). Using MC-Fold’s estimation ϵ of the energy contribution of forming the pair (*s*_5_, *t*_4_) given the state of its neighbors, the pairing is accepted or rejected depending on a relation between ϵ and a sampled random number $\xi \sim \mathcal {U}(0, 1)$. If $\xi \,<\, \sigma \left(\frac{-\varepsilon }{RT}\right)$, the base pair is accepted. *Middle:* The pair (*s*_5_, *t*_4_) is added to the structure. If $\xi \ge \sigma \left(\frac{-\varepsilon }{RT}\right)$ like for (*s*_6_, *t*_6_), then the pairing is rejected. *Bottom:* The pair (*s*_6_, *t*_6_) is removed from the structure. The structure may stay the same from one iteration to another if we accept an existing base pair or if we reject a candidate base pair. Neighbors may change over the course of the algorithm as only the direct neighboring base pairs are considered neighbors.

Figure 1 and Algorithm 1 depict the gist of this sampling process along with some specific details. Notably, we set the length *T* of the simulation to be proportional to the square of the number of nucleotides in the duplex, the proportionality factor ℓ being a hyperparameter that can be controlled by the user. Moreover, using a simulated annealing approach, we gradually reduce the temperature parameter *RT* at each iteration step, starting at *RT* = *u* and finishing at *RT* = $v$, both being editable hyperparameters. The random number generation (RNG) is done with MRG32k3a [45] and the random seed can be set by the user. Since the body of the loop is executed in $\mathcal {O}(n+m)$ time (proportional to the number of base pairs in the duplex), the overall time complexity of this sampling process is $\mathcal {O}\left((n+m)^3\right)$.


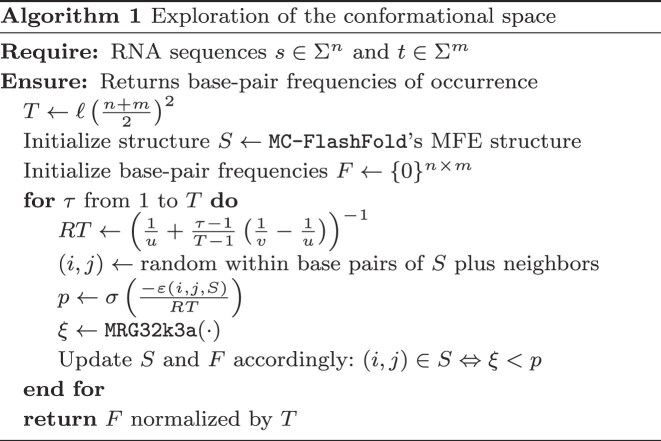


Once the simulation is complete, we can return a single structure that represents all structures visited during the simulation. In reality, we run multiple simulations to reduce variance and further explore the conformational space, the number of simulations being another hyperparameter. The simulations are independent from each other so we can exploit parallelism to execute them simultaneously. To return a consensus structure, we compute a maximum expected accuracy (MEA) structure based on the base-pair occurrences during the simulations. Having computed a frequency matrix *F* ∈ [0, 1]^*n* × *m*^, we can exploit dynamic programming to compute a structure that maximizes the sum of these base-pair frequencies. Typically, we compute the following optimal structure S*, where hyperparameter α is a threshold that allows to ignore low frequency base pairs [46], but more importantly, to control the precision-recall tradeoff as depicted in Supplementary Fig. S2. In short, the predicted structure is a MEA structure over a distribution of structures neighboring the MFE structure, with probabilities corresponding to frequencies of occurrence during the simulation.


\begin{eqnarray*}
S^* \in {argmax}_{S \in \mathcal {S}} \sum _{(i, j) \in S}F_{ij} {\mathbb{1}}\left\lbrace F_{ij} \ge \alpha \right\rbrace
\end{eqnarray*}

Since the frequencies *F*_*ij*_ are obtained through a finite number of sampling steps, the frequency matrix *F* consists in an approximation of the base-pairing probability map that could be computed by exhaustive exploration of the complete structure ensemble. This frequency matrix can be used to assess the stability of base pairs and mcdf can display the most frequent structures over the simulation along with their frequencies. In addition, mcdf provides two structural activity statistics: the frequency of transition *f* ∈ [0, 1] and an entropy estimation $e \in \mathbb {R}_{+}$. Respectively, these are the fraction of simulation steps where the duplex structure changes and the entropy of the Boltzmann distribution over the 500 most stable structures according to MC-FlashFold. Finally, mcdf returns the consensus structure S* for base-pair prediction.

### Datasets, performance measures, and algorithms

In May 2024, we selected structures from the PDB that were only composed of a single RNA molecule (no complexes) excluding G-quadruplexes or synthetic constructs. We solely selected structures determined experimentally by nuclear magnetic resonance or X-ray diffraction with a refinement resolution of at most 3.0 Å and we replaced modified nucleotides by their closest canonical ones. Since the ϵ parameters of MC-FlashFold and mcdf were determined with statistical inference over 531 PDB structures, we proceeded to split the selected structures into a training and a testing set.

To take into account both sequence and structural similarity while making the split, we used the RNA3DB dataset [47]. We first identified the components of RNA3DB that contained a structure that had at least 80% sequence similarity with a structure seen by MC-Fold. We identified eight such components: {1, 2, 4, 5, 8, 14, 26, 80}. We considered that these eight components were seen in their entirety by mcdf. After this step, the number of structures considered to be seen by mcdf increased from 531 to 1687. Then, within our selected structures from the PDB, a structure would be classified in the training set if it had at least 80% similarity with one of the 1687 sequences present in one of these eight components. Finally, we removed the redundancy among the training and testing sets by making sure that within each set, each pair of molecules had at most 80% sequence similarity. In the end, we obtained two nonredundant structurally dissimilar datasets composed of 154 structures in the training set and 162 structures in the testing set. The PDB identifiers of these structures are given in the supplementary data.

To identify base pairs, each structure was annotated using MC-Annotate [40]. From the output of MC-Annotate, stems were built by merging fragments of consecutive base pairs with at least one canonical base pair. We isolated these stems from their global structures and considered them as RNA duplexes so as to be inputs for RNA duplex secondary structure prediction tools. We ended up with 189 duplexes in the training set and 200 duplexes in the testing set by selecting the duplexes with at least 6 base pairs. The duplex sequences and secondary structures are provided in the supplementary data.

Note that these training and testing designations are mostly meant to indicate the similarity between the datasets’ structures and those that were seen by the MC-tools. This training set did not serve to determine MC-Fold’s parameters, but it did serve for tuning mcdf’s hyperparameters as described in the supplementary data. Meanwhile, to properly compare the generalization abilities of mcdf and MXfold2 [48], we constituted a separate training dataset for MXfold2 by selecting structures within the whole set of 1687 structures considered to be seen by MC-Fold, again removing non-RNA complexes and G-quadruplexes. To prevent MXfold2 from overfitting, we applied enhanced regularization techniques and reduced the capacity of the model. Notably, we kept the dropout rate at 50%, applied early stopping by executing three training epochs and increased regularization factors *C*_1_ and *C*_2_ 5-fold to end up with 0.625 and 0.05, respectively [48]. We also reduced the embedding size from 64 to 16 and the number of MLP and LSTM units from 32 to 8.

That being said, since we are measuring the performances of a variety of RNA 2D structure prediction algorithms on a base-pair detection task, our interest lies in true positives (*TP*), false positives (*FP*), and false negatives (*FN*). To compute those, we compare a predicted set of base pairs *S* with a ground truth set of base pairs *T*. In fact, we are especially interested in the precision (*P*), which is the fraction of predicted base pairs that are correct, and the recall (*R*), which is the fraction of ground truth base pairs that were properly predicted. In the case we are interested with a single measure of performance, we can compute the F-score (*F*), which is the harmonic mean between precision and recall.


\begin{eqnarray*}
TP = \vert S \cap T\vert \qquad FP = \vert S \setminus T\vert \qquad FN = \vert T \setminus S\vert
\end{eqnarray*}

\begin{eqnarray*}
P = \frac{TP}{TP + FP} \qquad R = \frac{TP}{TP + FN} \qquad F = \frac{2\cdot P\cdot R}{P+R}
\end{eqnarray*}

Now, within each dataset, we can report these scores by taking the mean over all structures, or we can measure the global scores on the whole dataset. The former provides us with the opportunity to estimate the variance of a performance score within a dataset, but the mean scores tend to be overestimated as RNA structure prediction algorithms perform better on small structures which are more frequent. Such performance measures are reported in next section for a variety of RNA 2D structure prediction algorithms. We computed performances for duplex-oriented methods on the duplex datasets composed of 189 and 200 duplexes, but we also computed performances for general structure prediction methods on the single-stranded datasets composed of 154 and 162 structures.

For duplex-oriented methods, the selected algorithms are mainly composed of RNA:RNA interaction prediction methods based on thermodynamics. To give a brief overview, for the general case where we allow for intramolecular base pairs between two RNA sequences of length *n* and *m*, RNAcofold [34] concatenates two RNA sequences and computes MFE structures like RNAfold [49] in $\mathcal {O}\left((n+m)^3\right)$. Simplifying the thermodynamic model by forbidding intramolecular interactions, DuplexFold [50] and RNAduplex [51] compute MFE duplex structures typically in $\mathcal {O}\left(n^2m^2\right)$, but this time complexity can be reduced to $\mathcal {O}\left(L^2nm\right)$ where *L* is the maximal length of an interior loop. Using an even more simplified thermodynamic model, RNAplex [52, 53] and RIsearch [54] allow for fast computation of approximate MFE structures in $\mathcal {O}\left(nm\right)$. We also measured the performances of RNAup [55] which takes into account the energetic cost of unpairing local structures. Finally, we considered LinearCoFold and LinearCoPartition [56] which concatenate sequences like RNAcofold before computing approximate MFE and MEA structures, respectively, in $\mathcal {O}\left(n + m\right)$. The Zipper algorithm acts as a lower bound on attainable performance scores as it simply returns the structure {(*i*, *i*) | 1 ≤ *i* ≤ min {*n*, *m*}}.

Incidentally, we are interested in the performances of algorithms that predict single-stranded RNA structures in a MEA or centroid fashion. We thus computed performances for Sfold [57, 58], CentroidFold [27], LinearPartition [59], and IPknot [46]. For general structure prediction, we also computed the performances of RNAfold, MC-FlashFold, LinearFold [60], and MXfold2, trained as discussed earlier. Since MC-FlashFold and Sfold can manage constraints that force the prediction of a duplex structure, they were included in the duplex benchmark as well. All algorithms in the duplex benchmark are forced to return a duplex structure without intramolecular interactions, except for RNAcofold, LinearCoFold, and LinearCoPartition. Still, only twice did LinearCoPartition predict intramolecular interactions in these datasets, in which case we returned the empty structure.

As described with Supplementary Tables S1 and S2, MC-DuplexFold (default) executes 100 simulations with ℓ = 10, α = 0.45, (*u*, $v$) = (3, 1/3), and an RNG seed of 1. In contrast, MC-DuplexFold (no init) naively initiates the simulations with the singleton structure {(*s*_*n*_, *t*_*m*_)} using α = 0.2. All other algorithms are set to default parameters except for MXfold2 which was retrained as described earlier and CentroidFold for which we activated the prediction of noncanonical base pairs using the CONTRAfold probability distribution [61]. All computations were done on a 13th Gen Intel(R) Core(TM) i7-1355U with 16 GB of RAM.

## Results and discussion

### Base-pair detection benchmarks

We are interested in precision, recall and computing time with respect to sequence length. We first report the mean and global scores for mcdf in comparison with other RNA duplex structure prediction algorithms in Table 1. These algorithms are organized in order of global F-score over all base pairs within the duplex testing dataset, which is composed of 2010 canonical and 220 noncanonical base pairs.

**Table 1.** Performances on duplex training dataset (top) and duplex testing dataset (bottom). Mean scores over each dataset are reported along with their standard deviation in a plus or minus fashion. The best scores are in bold for each dataset (ignoring Zipper’s computation time)

Structure	Mean *P*	Mean *R*	Global *P*	Global *R*	Global *F*	Total time (s)
MC-DuplexFold (no init)	0.938 ± 0.105	0.949 ± 0.097	0.922	0.932	0.927	1.880
MC-DuplexFold (default)	0.952 ± 0.100	0.954 ± 0.093	0.935	0.936	**0.936**	6.037
MC-FlashFold	0.942 ± 0.114	**0.965 ± 0.083**	0.919	**0.954**	**0.936**	0.653
Sfold	**0.972 ± 0.068**	0.850 ± 0.146	**0.965**	0.830	0.892	295.974
RIsearch	**0.972** ± 0.080	0.845 ± 0.154	0.962	0.824	0.887	**0.222**
RNAcofold	0.952 ± 0.148	0.828 ± 0.181	0.955	0.811	0.877	0.385
RNAduplex	0.950 ± 0.149	0.825 ± 0.180	0.952	0.809	0.875	0.293
RNAplex	0.950 ± 0.149	0.825 ± 0.180	0.952	0.809	0.875	0.306
LinearCoFold	0.950 ± 0.149	0.825 ± 0.180	0.953	0.808	0.874	3.076
LinearCoPartition	0.944 ± 0.175	0.817 ± 0.201	0.960	0.804	0.875	4.791
DuplexFold	0.941 ± 0.155	0.825 ± 0.183	0.940	0.809	0.870	4.205
RNAup	0.874 ± 0.284	0.667 ± 0.255	0.916	0.633	0.749	0.697
Zipper	0.758 ± 0.289	0.779 ± 0.283	0.680	0.713	0.696	0.001
MC-DuplexFold (no init)	0.947 ± 0.087	0.956 ± 0.083	0.934	0.941	0.938	2.254
MC-DuplexFold (default)	0.953 ± 0.093	0.957 ± 0.084	0.941	0.945	**0.943**	6.285
MC-FlashFold	0.942 ± 0.105	**0.966 ± 0.081**	0.922	**0.956**	0.939	0.702
Sfold	**0.981 ± 0.047**	0.881 ± 0.101	**0.975**	0.868	0.918	334.365
RIsearch	0.979 ± 0.055	0.864 ± 0.110	0.969	0.849	0.905	**0.235**
RNAcofold	0.973 ± 0.095	0.854 ± 0.138	0.967	0.848	0.903	0.405
RNAduplex	0.972 ± 0.093	0.854 ± 0.137	0.966	0.847	0.902	0.311
RNAplex	0.972 ± 0.093	0.854 ± 0.137	0.966	0.847	0.902	0.321
LinearCoFold	0.972 ± 0.093	0.854 ± 0.137	0.966	0.847	0.902	3.220
LinearCoPartition	0.962 ± 0.133	0.843 ± 0.159	0.965	0.840	0.899	3.310
DuplexFold	0.963 ± 0.108	0.846 ± 0.148	0.955	0.838	0.893	4.418
RNAup	0.896 ± 0.267	0.693 ± 0.237	0.947	0.678	0.791	0.787
Zipper	0.694 ± 0.322	0.713 ± 0.321	0.628	0.655	0.641	0.001

First of all, all algorithms perform curiously better on the testing set than on the training set, both on precision and recall, except for Zipper. Yet, the mean number of nucleotides per duplex is 21.83 for the training set versus 24.16 for the testing set. It seems the structures in the testing set must be, in general, better suited for thermodynamics-based prediction than those in the training set. Surprisingly, even MC-DuplexFold and MC-FlashFold perform better on the testing set than on the training set which provides evidence that the statistical learning of the MC-algorithms is not prone to overfitting. Still, their performance gain is less important than that of other algorithms, but justifiable by their already high scores on the training set. Comparing MC-DuplexFold and MC-FlashFold, we can notice the goal of balancing the precision-recall tradeoff has been accomplished.

In general, we can notice that solely predicting canonical base pairs yields better precision but lower recall, with Sfold and RIsearch having the best precisions and the MC-algorithms having the best recalls. Focusing on the MFE-based algorithms, we can notice that the heuristic approaches like LinearCoFold and RIsearch present equal or slightly better performances in comparison with exact MFE computation methods like RNAcofold or DuplexFold. This behavior supports previously reported results claiming that the use of large computational resources to calculate an exact optimal solution to the thermodynamic model may be counter-productive to accurately predict RNA structures [54, 60]. On top of that, using approximate or heuristic algorithms in the thermodynamic model has the advantage of speeding up the computation time. That is the case here for RIsearch, but not for the Linear-algorithms as they are meant to quickly infer structures for long sequences, which appears to come at the cost of a significative calculation constant overhead.

Since only mcdf and MC-FlashFold can predict noncanonical base pairs within the selected algorithms, we also measured the performances by ignoring noncanonical base pairs to better compare base-pair prediction abilities. The results are reported in Table 2. Again, Sfold and MC-FlashFold present the best precision and recall respectively, but the gaps have now decreased. Looking at the testing set, Sfold now yields the best F-score, closely followed by MC-DuplexFold and MC-FlashFold. This highlights the benefits of structure sampling, but also suggests that considering noncanonical base pairs may help in predicting canonical base pairs, albeit minimally (∼3% mean gain in global recall).

**Table 2.** Performances on duplex training dataset (top) and duplex testing dataset (bottom) for AU, GC, and GU base pairs only. Mean scores over each dataset are reported along with their standard deviation in a plus or minus fashion. The best scores are in bold for each dataset

Structure	Mean *P*	Mean *R*	Global *P*	Global *R*	Global *F*
MC-DuplexFold (no init)	0.958 ± 0.080	0.965 ± 0.083	0.948	0.951	0.950
MC-DuplexFold (default)	0.968 ± 0.075	0.968 ± 0.078	0.960	0.954	0.957
MC-FlashFold	0.964 ± 0.082	**0.975 ± 0.073**	0.954	**0.965**	**0.959**
Sfold	**0.972 ± 0.068**	0.954 ± 0.088	**0.965**	0.946	0.956
RIsearch	**0.972** ± 0.080	0.947 ± 0.104	0.962	0.939	0.950
RNAcofold	0.952 ± 0.148	0.925 ± 0.160	0.955	0.925	0.939
RNAduplex	0.950 ± 0.149	0.923 ± 0.160	0.952	0.922	0.937
RNAplex	0.950 ± 0.149	0.923 ± 0.160	0.952	0.922	0.937
LinearCoFold	0.950 ± 0.149	0.922 ± 0.160	0.953	0.921	0.937
LinearCoPartition	0.944 ± 0.175	0.914 ± 0.185	0.960	0.917	0.938
DuplexFold	0.941 ± 0.155	0.923 ± 0.163	0.940	0.923	0.931
RNAup	0.874 ± 0.284	0.745 ± 0.275	0.916	0.722	0.807
Zipper	0.848 ± 0.217	0.780 ± 0.286	0.837	0.715	0.771
MC-DuplexFold (no init)	0.968 ± 0.061	0.970 ± 0.072	0.961	0.959	0.960
MC-DuplexFold (default)	0.973 ± 0.067	0.972 ± 0.072	0.968	0.963	0.965
MC-FlashFold	0.969 ± 0.072	**0.977** ± 0.069	0.960	**0.971**	0.965
Sfold	**0.981 ± 0.047**	0.971 **± 0.060**	**0.975**	0.963	**0.969**
RIsearch	0.979 ± 0.055	0.954 ± 0.085	0.969	0.942	0.956
RNAcofold	0.973 ± 0.095	0.941 ± 0.122	0.967	0.940	0.953
RNAduplex	0.972 ± 0.093	0.941 ± 0.121	0.966	0.939	0.952
RNAplex	0.972 ± 0.093	0.941 ± 0.121	0.966	0.939	0.952
LinearCoFold	0.972 ± 0.093	0.941 ± 0.121	0.966	0.939	0.952
LinearCoPartition	0.962 ± 0.133	0.929 ± 0.151	0.965	0.932	0.949
DuplexFold	0.963 ± 0.108	0.932 ± 0.134	0.955	0.930	0.942
RNAup	0.896 ± 0.267	0.767 ± 0.259	0.947	0.753	0.839
Zipper	0.805 ± 0.255	0.706 ± 0.330	0.810	0.643	0.717

**Figure 2.**
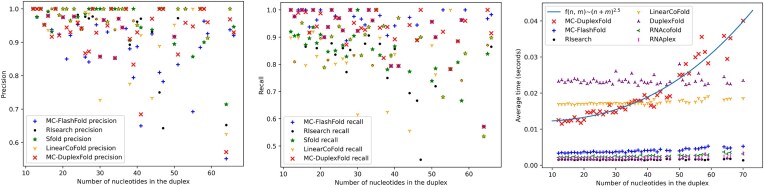
Distributions for precision (left), recall (middle), and computation time (right) over sequence lengths. Performance scores computed on testing dataset. Execution times computed on training and testing datasets combined. When multiple duplexes have the same number of nucleotides, the mean value is reported.

Looking at the influence of sequence length on the predictions, Fig. 2 depicts the precision and recall distributions over sequence lengths for some algorithms on the testing set. We can notice how Sfold and RIsearch tend to have better precision than recall, and conversely for MC-FlashFold. In all cases, performances deteriorate with the length of the duplex, but still, these algorithms can attain near-perfect precision or recall on the biggest sequences in this dataset.

Regarding the influence of sequence length on execution times, Fig. 2 shows how sequence length has little influence over the computation times of most algorithms, except for mcdf which is significantly impacted by the number of nucleotides in the duplex. Again, mcdf is meant to study the dynamics of local interactions after a global scan has been executed. As it happens, mcdf only tolerates sequences with <100 nucleotides (200 total in the duplex), which requires a computation time of about half a second with the default parameters.

The default initiation with MC-FlashFold’s MFE structure yields similar but slightly better results than MC-DuplexFold (no init), which is relevant for simulating base-pair propagation along RNA duplexes. However, mcdf’s simulations can be initialized with any secondary structure, which can moderately alter the portion of the conformational space that is explored by mcdf. For instance, in a situation where a duplex secondary structure is first inferred from sequence with canonical base pairs only, one can use mcdf to explore neighboring structures and output a representative MEA structure which can contain noncanonical base pairs while potentially correcting initially predicted canonical base pairs. Table 3 presents the performances of mcdf when initialized with the structures predicted by all algorithms.

**Table 3.** Performances for all base pairs (top) and AU, GC, and GU base pairs only (bottom) on duplex testing dataset for mcdf when initialized with all algorithms. Mean scores over the dataset are reported along with their standard deviation in a plus or minus fashion. The best scores are in bold for each dataset. The last column reports the difference between the global F-score measured when initiating mcdf with each algorithm versus the global F-score measured for each algorithm alone

Initiation	Mean *P*	Mean *R*	Global *P*	Global *R*	Global *F*	Global *F* gain
MC-DuplexFold	0.953 ± 0.090	0.956 ± 0.089	0.941	0.942	0.942	-0.001
MC-FlashFold	0.953 ± 0.093	0.957 ± 0.084	0.941	0.945	0.943	0.004
Sfold	0.965 **± 0.065**	**0.960 ± 0.076**	0.956	**0.946**	**0.951**	0.033
RIsearch	**0.966** ± 0.066	0.957 ± 0.090	**0.958**	0.940	0.949	0.044
RNAcofold	0.965 ± 0.069	0.953 ± 0.095	0.956	0.940	0.948	0.045
RNAduplex	0.965 ± 0.068	0.954 ± 0.094	0.957	0.941	0.949	0.047
RNAplex	0.965 ± 0.068	0.954 ± 0.094	0.957	0.941	0.949	0.047
LinearCoFold	0.965 ± 0.068	0.954 ± 0.094	0.957	0.941	0.949	0.047
LinearCoPartition	0.963 ± 0.069	0.952 ± 0.096	0.954	0.937	0.945	0.046
DuplexFold	0.964 ± 0.076	0.947 ± 0.111	0.955	0.930	0.943	0.050
RNAup	0.953 ± 0.094	0.914 ± 0.158	0.945	0.892	0.918	**0.127**
Zipper	0.912 ± 0.173	0.853 ± 0.226	0.907	0.823	0.863	0.222
MC-DuplexFold	0.973 ± 0.065	0.971 ± 0.076	0.967	0.961	0.964	-0.001
MC-FlashFold	0.973 ± 0.067	0.972 ± 0.072	0.968	0.963	0.965	0.000
Sfold	0.981 **± 0.042**	**0.977 ± 0.056**	0.977	**0.968**	**0.973**	0.004
RIsearch	**0.983** ± 0.044	0.973 ± 0.074	**0.978**	0.961	0.969	0.013
RNAcofold	0.980 ± 0.049	0.970 ± 0.078	0.975	0.961	0.968	0.015
RNAduplex	0.981 ± 0.048	0.971 ± 0.077	0.976	0.962	0.969	0.017
RNAplex	0.981 ± 0.048	0.971 ± 0.077	0.976	0.962	0.969	0.017
LinearCoFold	0.981 ± 0.048	0.971 ± 0.077	0.976	0.962	0.969	0.017
LinearCoPartition	0.979 ± 0.049	0.968 ± 0.079	0.974	0.959	0.966	0.017
DuplexFold	0.979 ± 0.059	0.964 ± 0.096	0.974	0.952	0.963	0.021
RNAup	0.969 ± 0.081	0.932 ± 0.140	0.964	0.916	0.939	**0.100**
Zipper	0.934 ± 0.153	0.864 ± 0.226	0.938	0.837	0.885	0.168

As mcdf is initialized with MC-FlashFold by default, the MC-FlashFold lines simply repeat the mcdf lines from previous tables. The mcdf lines, on the opposite, initiate mcdf with the default output of mcdf. In this case, we can notice the performances are practically the same, which suggests that executing mcdf recursively is not of particular interest. For the other algorithms, however, the performances are systematically increased when mcdf is executed afterwards, not only for noncanonical base pairs, but for canonical base pairs as well. This reaffirms how taking noncanonical base pairs into account can benefit the prediction of canonical base pairs. Especially, it happens that the precision on canonical base pairs is improved for general algorithms when mcdf is executed afterwards. This result highlights one of the advantages of initiating a simulation at a pre-inferred structure and computing an MEA structure (in comparison to supplying the pre-inferred structure as strict structural constraints for canonical base pairs), namely that false positives in the initial prediction can be corrected. Table 5 portrays two such examples. As a matter of fact, initiating the simulations of mcdf with all canonical-only algorithms (from Sfold to RNAup in the tables) results in an average global precision increase, whereas fixing the canonical base pairs as strict structural constraints to MC-FlashFold has the opposite effect.

**Table 4.** Performances for all base pairs on single-stranded testing dataset without pseudoknots. Results are reported for all algorithms (top) and for mcdf when initialized with all algorithms (bottom). Mean scores are reported along with their standard deviation in a plus or minus fashion. The best scores are in bold for each dataset (ignoring Zipper’s computation time). The last column reports the total computation time (top) and the difference between the global F-score measured when initiating mcdf with each algorithm versus the global F-score measured for each algorithm alone (bottom)

Initiation	Mean *P*	Mean *R*	Global *P*	Global *R*	Global *F*	Total time/*F* gain
Sfold	**0.939 ± 0.151**	0.836 **± 0.149**	**0.934**	0.814	**0.870**	219.807
RNAfold	0.912 ± 0.188	0.825 ± 0.182	0.899	0.801	0.847	**0.324**
LinearPartition	0.916 ± 0.169	0.813 ± 0.170	0.903	0.788	0.842	3.392
MXfold2	0.912 ± 0.190	0.816 ± 0.180	0.896	0.791	0.840	96.660
CentroidFold	0.844 ± 0.155	0.880 **± 0.149**	0.816	**0.856**	0.835	1.291
IPknot	0.918 ± 0.189	0.795 ± 0.195	0.918	0.760	0.831	242.245
MC-DuplexFold++	0.829 ± 0.185	0.879 ± 0.173	0.790	0.842	0.816	7.547
MC-FlashFold	0.814 ± 0.189	**0.885** ± 0.173	0.770	0.851	0.809	0.998
LinearFold	0.870 ± 0.260	0.755 ± 0.252	0.890	0.734	0.804	3.207
Zipper	0.580 ± 0.342	0.604 ± 0.337	0.428	0.483	0.454	0.001
Sfold	**0.921 ± 0.155**	0.868 **± 0.147**	**0.912**	**0.851**	**0.880**	**0.010**
RNAfold	0.893 ± 0.189	0.845 ± 0.188	0.881	0.822	0.850	0.003
LinearPartition	0.906 ± 0.167	0.836 ± 0.175	0.890	0.810	0.848	0.006
MXfold2	0.894 ± 0.192	0.838 ± 0.187	0.878	0.814	0.845	0.005
CentroidFold	0.859 **± 0.155**	0.863 ± 0.164	0.842	0.834	0.838	0.003
IPknot	0.908 ± 0.187	0.818 ± 0.198	0.905	0.780	0.838	0.007
MC-DuplexFold++	0.828 ± 0.185	0.877 ± 0.174	0.791	0.841	0.815	-0.001
MC-FlashFold	0.829 ± 0.185	**0.879** ± 0.173	0.790	0.842	0.816	0.007
LinearFold	0.854 ± 0.258	0.776 ± 0.260	0.870	0.755	0.808	0.004
Zipper	0.726 ± 0.295	0.641 ± 0.318	0.718	0.532	0.611	0.157

**Table 5.** Correction of false positives by mcdf. The threshold α is set to 0 to increase the false-positive rate for mcdf. True positives are depicted in green; and false positives in red. Canonical base pairs are represented with bars; and noncanonical base pairs with colons

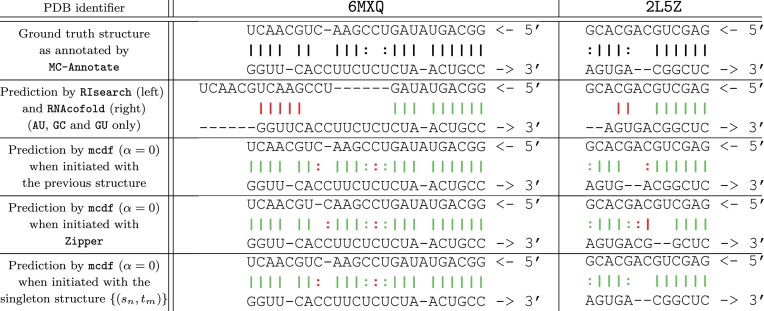

As mcdf’s simulations can be initialized with any duplex secondary structure, we can apply mcdf to general structure prediction as well. Namely, provided a secondary structure prediction for a single-stranded RNA sequence *s*, we can apply mcdf to each stem in a predicted secondary structure. We denote this process by the algorithm MC-DuplexFold++ which builds upon MC-FlashFold’s MFE prediction by default. Table 4 reports the performances for general structure prediction algorithms on the single-stranded testing dataset from which we removed the pseudoknotted structures. The algorithms are ordered by global F-score computed on all base pairs.

Looking at the predictions of each algorithm individually, we notice Sfold and MC-FlashFold still present high precision and recall, respectively, while the best global recall is now held by CentroidFold. As Sfold particularly presents low variance in its performances, it holds the best F-score despite having lower recall than CentroidFold. Although the performances held by Sfold, LinearPartition, and MXfold2 provide evidence that heuristic and approximate methods constitute a viable alternative to exact free-energy minimization methods, the quality of RNAfold’s predictions underscore the continued relevance of exact MFE structure computation. Note that we turned to the bioconda implementation of Sfold, used the web services for IPknot and did not rely on a GPU for MXfold2’s computations, which may explain their important computation times. Concerning overfitting for MXfold2, due to the enhanced regularization and reduced capacity described earlier, no overfitting behavior is observable in this benchmark. In fact, MXfold2’s performances are better on the testing set than on the training set, just like other algorithms. However, that is not the case when MXfold2 is trained with its default training and architecture parameters.

Now, regarding mcdf’s ability to refine predictions from other algorithms, we again observe a global F-score increase, but this is mostly due to the addition of noncanonical base pairs to the initial predictions. The performances remain relatively the same when we only consider canonical base pairs. Also, mcdf can be applied to any predicted stem in a global RNA secondary structure, which includes pseudoknots. However, it cannot help in the arduous task of identifying the location of said pseudoknots. Hence, as evidenced by Supplementary Table S3, the use of mcdf++ does not generally improve performances for pseudoknotted structures. Actually, the opposite effect can be observed as mcdf++ refines the predictions of each predicted stem, including wrongly identified ones. Indeed, refining a wrongly predicted stem of canonical base pairs generally results in adding false-positive noncanonical base pairs to the initial prediction which results in a global precision decrease. Altogether, these results highlight an important limitation of mcdf, namely that mcdf should mostly be used to study the structural dynamics of already identified stems or RNA:RNA interactions, and not to predict their location.

### Study of divergence

mcdf’s ability to correct false-positive predictions from other algorithms depends on its propensity to explore the conformational space and diverge from its initial structure. Looking at Tables 2 and 3, we can indeed expect mcdf to explore various structures as RIsearch’s global precision on the canonical base pairs of the testing set increases from 0.969 to 0.978 when RIsearch’s prediction is used to initialize mcdf’s simulations. Table 5 highlights two specific examples where false-positive canonical base pairs from other algorithms are corrected by mcdf.

To make sure false positives are indeed replaced by true positives and not only removed from the initial structure, we set mcdf’s hyperparameter α to zero, which maximizes the number of base pairs predicted by mcdf. Studying PDB structures 6MXQ and 2L5Z, we can indeed observe that false-positive predictions from other algorithms can be replaced by true positives through the use of mcdf. Other false positives are still present after mcdf’s refinement, but they do not affect the global shape of the ground truth structure as they mainly consist in opportunistic interactions caused by the zero-valued threshold α. Looking at mcdf’s predictions when initiated with Zipper or the singleton structure {(*s*_*n*_, *t*_*m*_)}, we can observe that the final structure returned by mcdf is only moderately affected by the initial structure, just as Table 3 suggested. In particular, the Zipper initiation favors base pairs that are face-to-face with respect to their positioning within the two interacting strands.

Further analyzing mcdf’s tendency to explore the conformational space neighboring its initial structure, we studied the hundred structures where mcdf’s simulations ended when provided with RIsearch’s prediction for 6MXQ. Among these simulations, we obtained 65 different final structures. For each one, a distance to the initial prediction was computed as the number of base pairs that need to be removed plus the number of base pairs that need to be added to get from one structure to another. We computed a minimum distance of 2 and a maximum distance of 33 with a mean distance of 15.42. Thus, mcdf does not simply confirm the initial prediction, but it rather explores the conformational space with the ability to escape local minima.

**Figure 3.**
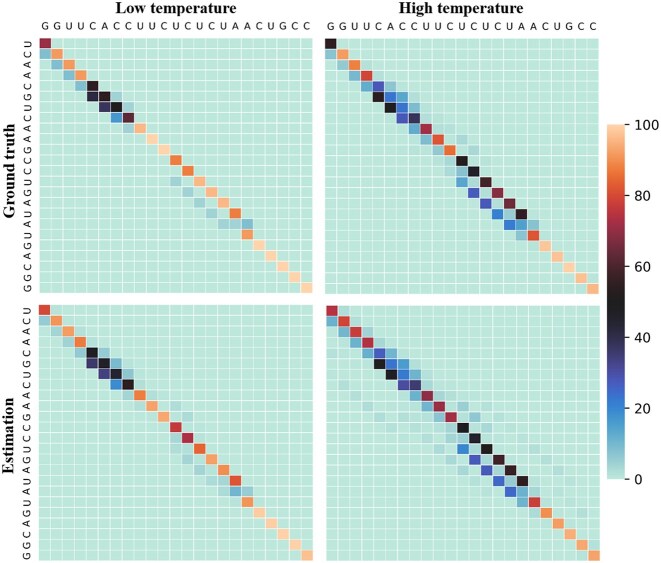
Comparison between probabilities estimated by mcdf and ground truth probabilities computed with partial partition functions for 6MXQ. Ground truth probabilities are determined with a partition function computed on the portion of the conformational space with a free energy below a certain threshold according to MC-FlashFold. We used thresholds that correspond to 94% (upper left) and 82% (upper right) of the MFE. Probabilities were estimated using mcdf with $u = \frac{1}{v} = 1$ (lower left) and $u = \frac{1}{v} = 3$ (lower right).

Regarding mcdf’s connection with the Boltzmann distribution from which it is sampling, we noted earlier that the normalized frequency matrix *F* computed by mcdf consists in an approximation of the true probabilities that could be computed using a complete partition function. To verify if the approximated probabilities are consistent with ground truth probabilities, we compared mcdf’s approximations with probabilities computed by partial partition functions on 6MXQ again. Namely, to account for the different temperature parameters that are permitted by mcdf, the ground truth probabilities were determined with partial partition functions that only consider portions of the conformational space with structures that have a free energy below a certain threshold. We used thresholds that correspond to 94% and 82% of the MFE and compared them to probabilities estimated by mcdf with $u = \frac{1}{v} = 1$ (no simulated annealing) and $u = \frac{1}{v} = 3$ (default parameters), respectively. Figure 3 visually depicts the resemblance between estimated and ground truth probabilities. These probabilities almost perfectly correlate (*R*^2^ = 0.99), and they still highly correlate when we ignore the probabilities below 0.01 (*R*^2^ = 0.98). In sum, this section provides evidence that mcdf has the ability to adequately explore the conformational space, escape local minima, sample from expected distributions and correct false positives from initial predictions.

### MiRNA:mRNA duplexes and miR-34a:Sirt1

The formation of miRNA:mRNA duplexes within the RISC is hierarchical [19, 32]. Base pairing typically initiates with the miRNA’s nucleotides 2–5 before spreading to nucleotides 6–8, in which case a conformational shift in the helix-7 of the Argonaute protein allows for pairing in the supplementary region (nucleotides 13–17) and then the whole duplex [62]. The specifics of these dynamic interactions are still poorly understood.

Experimental validation of miRNA:target interactions requires a protocol that integrates multiple approaches to capture both dominant and transient structural states. *R*_1ρ_ relaxation-dispersion NMR spectroscopy is essential for visualizing excited states, while MC-Fold structure predictions provide computational models of these transient conformations. Mutagenesis experiments then confirm which predicted structural states are actually adopted, as demonstrated in previous studies [31]. In contrast, RNA:RNA binding analysis using SHAPE (RABS) primarily reveals the dominant conformation and lacks the resolution to explore the dynamic nature of miRNA:target duplex formation [63].

**Figure 4.**
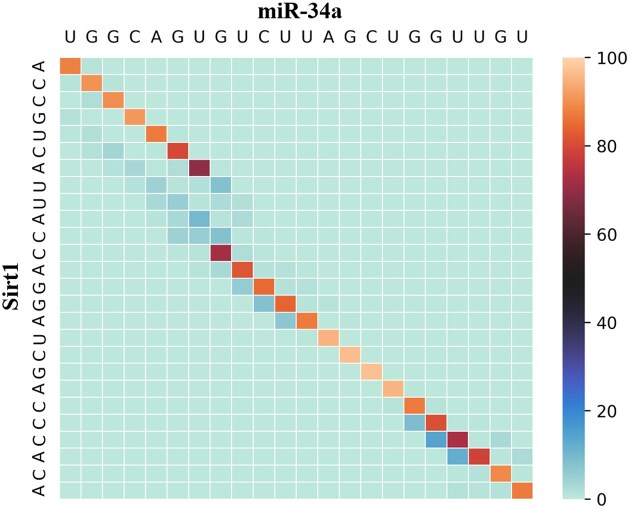
Estimations of base-pairing probabilities for the miR-34a:Sirt1 duplex. Normalized frequencies (in percentage) are computed by mcdf with default parameters.

In the case of miR-34a and Sirt1, it has been shown that the structure adopted by the duplex corresponds to a sub-optimal structure in the sense of thermodynamics optimization, which allows for the formation of a relatively strong 8mer seed type instead of a weaker 7mer-A1 seed type [31]. During duplex formation, the structure could undergo a coordination step in which the duplex would shift between the ground state and the excited state [64]. Using mcdf, we can simulate the behavior of the miR-34a:Sirt1 duplex to identify unstable base pairs. Figure 4 shows the frequencies of occurrence of each base pair when executed on mcdf with default parameters.

While the structure predicted by mcdf represents the ground state, the frequency table reveals regions that may be subject to structural dynamics. Notably, the single base-pair shift previously identified [31] is detectable in mcdf simulations. When simulations are executed without simulated annealing, the GU base pair of the excited state appears 12.4% of the time compared to 80.6% for the GC base pair of the ground state. Additionally, as mcdf provides the frequencies of occurrence for the most probable structures, we can observe that the ground state is the most frequent, appearing in 44.36% of simulation steps, while the excited state is the second most frequent, occurring 6.82% of the time. These results corroborate NMR and RABS experimental findings, further supporting the dynamic nature of RNA duplex formation and the presence of transient structural states.

### Mutations in miR-125a affect maturation

MiRNA genes are transcribed by RNA polymerase II into primary miRNAs (pri-miRNAs) which undergo various maturation steps before transforming into mature miRNAs [19]. A substructure of the pri-miRNA adopts a long hairpin structure which is processed by Drosha and DGCR8 proteins (the microprocessor) to release a shorter hairpin of about 22 base pairs, the precursor miRNA (pre-miRNA) [65]. This pre-miRNA is processed again by Dicer to cut-off the apical loop leaving a miRNA duplex from which one strand, the mature miRNA, will end up loaded in the RISC for target recognition [66]. Various factors influence maturation efficiency, including the sequence composition of the pri-miRNA.

For instance, in the case of miR-125a, it has been reported that a SNP could significantly disrupt the maturation pathway from pri-miRNA to pre-miRNA [20]. This case has been studied through multiple experiments [21, 67] and computational models have been established to predict maturation data from sequence [22]. The specifics of the phenomenon still need to be clarified.

In the context where the structural activity of the pri-miRNA has an impact on maturation, it should be possible to observe a correlation between maturation data and structural activity statistics. The latter can, for instance, be represented by the frequency of transition *f* ∈ [0, 1] provided by mcdf, that is, the fraction of simulation steps where the duplex structure shifts from one to another. In addition, mcdf can display entropy information $e \in \mathbb {R}_{+}$ by estimating the entropy of the Boltzmann distribution over the 500 most stable structures according to MC-FlashFold. With these two statistics in hand, we would like to predict maturation data.

**Figure 5.**
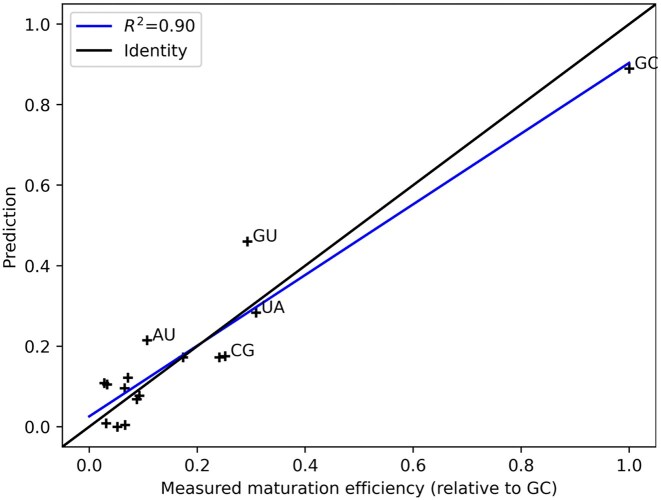
Correlation between experimental and predicted maturation efficiencies for miR-125a. Predictions are computed by Ridge regression from structural activity statistics returned by mcdf.

We therefore turn our attention to the maturation efficiencies of 16 miR-125a variants, all base pairs at the SNP position [67]. These experiments were conducted *in vivo*. We performed a simple linear regression to predict maturation from *e*, *f*, log *e*, and log *f*. Figure 5 shows how structural activity statistics calculated by mcdf can be used to predict maturation data. In contrast, when this method is used to predict maturation data measured *in vitro* [21], almost no predictive power is observed (*R*^2^ = 0.20). Note that the *in vivo* and *in vitro* measurements poorly correlate (*R*^2^ = 0.30).

### Prediction of equilibrium dissociation constants

Again looking at miRNA:mRNA interactions, target repression depends on the strength of the miRNA:mRNA interaction which, in terms of kinematics, can be described with a dissociation constant (*K*_d_) [68]. We thus attempted to predict *K*_d_ values from sequence via structural dynamics statistics computed by mcdf. We selected *K*_d_ values reported for thousands of target sequences for let-7a and miR-21 [69]. We first folded the duplexes using MC-FlashFold to compute MFE energies, and also folded the duplexes with mcdf to compute log *f* and *e* as described in last section. We did so for complete duplexes, but also for seed and supplementary regions separately. We were therefore provided with nine statistics from which to predict dissociation constants.

Considering the size of the selected datasets (11 107 and 8309 data points for let-7a and miR-21, respectively) and because miRNA:mRNA duplexes fold in a hierarchical manner [70], we proceeded to split both let-7a and miR-21 datasets into training (90%) and testing (10%) sets before training random forest regressor algorithms on the training sets using the scikit-learn package [71]. We computed correlation coefficients between the predicted and measured log *K*_d_ in the testing sets for let-7a (*R*^2^ = 0.75) and miR-21 (*R*^2^ = 0.55). We notably observed that mcdf’s predictive power resembles MC-FlashFold’s as we obtain correlation coefficients of *R*^2^ = 0.68 and 0.44 on let-7a and miR-21, respectively, when solely using mcdf’s statistics versus 0.69 and 0.46 when solely using MC-FlashFold’s MFE. Therefore, the statistics computed by mcdf should not be regarded as replacements for existing predictor features, but rather as complementary attributes that may improve predictive models by capturing aspects of structural dynamics.

In short, mcdf provides secondary structure activity statistics that can be used to model RNA duplex dynamics, predict pri-miRNA maturation data and predict miRNA:mRNA duplex dissociation constants. These statistics complement already available predictor features like target accessibility, conservation and kinetic data [72] and we recommend using them altogether.

## Conclusion

In conclusion, the prediction of RNA duplex secondary structure can benefit from considering noncanonical base pairs, modeling structural dynamics, exploring the conformational space and simplifying the thermodynamic model. As shown by Sfold’s performances in base-pair prediction benchmarks, sampling from the Boltzmann distribution seems to be particularly efficient in predicting intermolecular base-pair interactions in RNA:RNA hybrid structures. Besides, when sampling is done with MCMC simulations, secondary structure activity can be abstracted by base-pairing frequencies and structural activity statistics, which can subsequently be used as predictor features to model miRNA maturation data or miRNA:mRNA dissociation constants. Such is the spirit of MC-DuplexFold, which also identifies noncanonical and unstable base pairs, critical for assessing the specificity of RNA:RNA interactions.

Precisely, mcdf applies Gibbs sampling to form and break base pairs one at a time before returning a consensus MEA structure to represent the portion of the conformational space that has been explored during the simulations. This framework has the advantage of being convenient for incorporating predictions from other methods, as simulations can be initialized with any duplex secondary structure. Actually, benchmarking results show that mcdf generally improves base-pair prediction accuracy when used in conjunction with a panoply of well-established RNA 2D structure prediction algorithms, albeit minimally. Not only can noncanonical base pairs be added to canonical-only predictions, but false positives from the original predictions can be corrected.

Benchmarking results show that heuristic and approximate methods are particularly efficient for predicting intermolecular base pairs in RNA duplexes, suggesting inaccuracies in the thermodynamic model render exact optimization unnecessary. Still, general structure prediction benchmarks exhibit how exact MFE computation methods like RNAfold remain relevant. On a different note, we can observe that mcdf and MC-FlashFold are not prone to overfitting, whereas careful management of capacity and regularization is necessary to avoid overfitting behavior from MXfold2 which, among all deep learning-based RNA folding algorithms, seems in all likelihood to be the least inclined to suffer from overfitting [73]. Still, mcdf does not consider modified nucleotides, nor reactivity data, nor cofactors of the cellular environment, and its execution time is highly sensible to sequence length with a worst-time complexity in $\mathcal {O}((n+m)^3)$. Moreover, mcdf is not meant to identify the location of RNA:RNA interactions, but rather to study the structural dynamics of already identified duplexes. Hence, mcdf can deteriorate predictions when applied, for instance, on mistakenly located pseudoknots.

As RNA folding is increasingly valued in fundamental molecular biology research, the development of specialized RNA structure prediction tools becomes more and more important. Notably, assessing RNA duplex structures from sequence while taking into account noncanonical base pairs and structural dynamics is of particular interest. As mcdf provides such information reasonably quickly for RNA sequences of <100 nucleotides, it serves as a valuable tool for modeling miRNA:mRNA interactions and miRNA maturation efficiency. This work therefore consists in a forward step toward unraveling the mystery of miRNA activity, which paves the way to revolutionary breakthroughs in the fields of biology and medicine.

## Supplementary Material

lqaf099_Supplemental_Files

## Data Availability

The data underlying this article are available at: https://github.com/major-lab and https://figshare.com/articles/software/MC-DuplexFold_source_code/28608962?file=53048960.
