# Supplementary material for: Modeling RNA duplex dynamics with Gibbs sampling enhances base-pair prediction accuracy and reveals structural activity profiles
Source: NAR Genom Bioinform. 2025 Jul 17;7(3):lqaf099. doi: 10.1093/nargab/lqaf099 (PMC12267985; doi:10.1093/nargab/lqaf099)
Supplement: lqaf099_Supplemental_Files [file lqaf099_supplemental_files.zip › MC_DuplexFold_SuppMat.pdf]

Nucleic Acids Research, 2025, 1–3
doi: DOI HERE
Advance Access Publication Date: Day Month Year
Paper

Modeling RNA Duplex Dynamics with Gibbs Sampling Enhances Base-Pair Prediction Accuracy and Reveals Structural Activity Profiles

Simon Chasles and François Major*

Department of Computer Science and Operations Research, and Institute for Research in Immunology and Cancer, Université de Montréal, Montréal, Québec, Canada, H3C 3J7

*Corresponding author. francois.major@umontreal.ca

FOR PUBLISHER ONLY Received on Date Month Year; revised on Date Month Year; accepted on Date Month Year

Supplementary Data

Hyperparameter tuning

While the philosophy behind `mcdf` is to simulate the evolution of a structure through time, its effective behavior is to explore likely structures around a structure of interest, by default, `MC-FlashFold`'s MFE structure. We thus want the simulations to explore structures that are different from the initial starting point, which means we want the simulations to be long enough and to present a certain degree of randomness. By setting ℓ to 10 and (u, v) to $(2, 1/2)$, we tested the impact of the number N of simulations on precision and recall as reported in table 1, with α being set by default to 1/2. To account for variability in the results due to random number generation, the values reported in this section are mean values over 6 different seeds. All global scores are measured on the training set.

N	1	3	9	27	81	243	729
P	0.887	0.922	0.931	0.929	0.928	0.928	0.928
R	0.895	0.921	0.928	0.931	0.933	0.933	0.934
F	0.891	0.921	0.929	0.930	0.930	0.930	0.931

Supplementary table 1. Influence of number N of simulations over global precision and recall on the training set. Reported values are mean values over 6 different seeds for random number generation.

In general, we observe that as more simulations are executed, the recall is increased while the precision tends to reach a certain plateau around $N \approx 10$. Also, the more simulations we do, the less variance we have in the results. However, too big N makes the execution time too high. We selected $N = 100$ as default parameter as it gives a good balance between performance, variability and execution time. In the same manner, we tested ℓ for multiple values after fixing N to 100, as reported in table 2.

ℓ	2	4	7	11	18	29	47
P	0.917	0.920	0.923	0.930	0.939	0.943	0.945
R	0.945	0.942	0.938	0.932	0.924	0.919	0.915
F	0.931	0.931	0.931	0.931	0.931	0.931	0.930

Supplementary table 2. Influence of length ℓ of each simulation over global precision and recall on the training set. Reported values are mean values over 6 different seeds for random number generation.

The length ℓ of each simulation has a direct impact on the precision-recall tradeoff with low values of ℓ making the visited structures resemble `MC-FlashFold`'s MFE structure which means low precision and high recall. We thus wanted ℓ to be big enough, but not too big as it would affect both computation time and performances. We set $\ell = 10$ as default value with the intention to lower the computation time and to let the temperature parameters (u, v) impact the exploration of the conformational space. The philosophy is thus to set $v = 1/u$ with $u > 1$ to first introduce randomness in the structure exploration before sticking to a relatively stable structure. Figure 1 depicts how the pairing probabilities are influenced by the value of RT. A value of RT greater than one will aggregate probabilities around 1/2 introducing more randomness to the simulation. On the other hand, a value of RT smaller than one will cluster the probabilities around 0 or 1 depending on the sign of the estimated energy contribution.

We tested $u \in \{1, 1.5, 2, 3, 4, 6, 9\}$ and chose $u = 3$ as default value as it yielded the better performances in terms of F-score. Still, such a combination of hyperparameters led to a certain imbalance between precision and recall. To reduce the precision-recall divergence, we finally tuned pairing threshold α to the default value of 0.45, as justified by figure 2. Note that all these hyperparameters can be tuned by the user to match desired behaviors.

© The Author 2025. Published by Oxford University Press. All rights reserved. For permissions, please e-mail: journals.permissions@oup.com

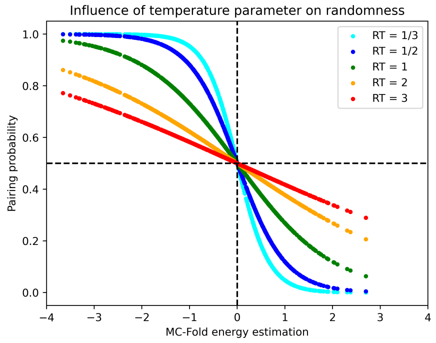

Supplementary figure 1: Influence of temperature parameter RT on pairing probabilities. The probabilities are only reported for `MC-Fold`'s energy estimations of 1-bulges and consecutive stacks.

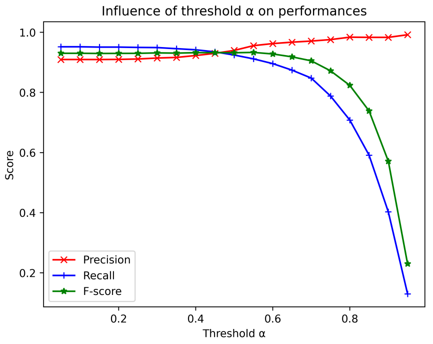

Supplementary figure 2: Influence of pairing threshold α on precision, recall and F-score on the training set for a single random number generation seed.

Number of duplex structures

Theorem 1 (Number of duplex structures) *Given two sequences s and t of length n and m respectively, the number $K(n,m)$ of possible duplex structures without intramolecular, multiple or crossing base pairs is* $\binom{n+m}{n} = \binom{n+m}{m}$.

Proof We proceed by induction on $n \geq 1$ for arbitrary $m \in \mathbb{N}$.

If $n = 1$, this single nucleotide of s can pair with either of the m nucleotides of t, or pair with none, which means there are $m+1$ possible structures. We thus have that $K(1,m) = m+1 = \binom{1+m}{1} = \binom{n+m}{n} = \binom{n+m}{m}$. This is true even if $m = 0$ since the empty set is the only possible structure when s hybridizes with the empty sequence.

Now, assume that $\exists n \in \mathbb{N} : \forall m \in \mathbb{N} : K(n,m) = \binom{n+m}{n} = \binom{n+m}{m}$ and consider $K(n+1,m)$ for arbitrary $m \in \mathbb{N}$. The total number of structures is the number of structures where s_{n+1} is unpaired plus the number of structures where s_{n+1} is paired. For the former, fixing s_{n+1} to be unpaired is equivalent to ignoring s_{n+1} which means we have $K(n,m)$ possible structures. For the latter, if s_{n+1} is paired to t_r with $r \in \{1,\ldots,m\}$, we have $K(n,r-1)$ possible structures since there are no crossing base pairs. We thus have the following relation.

$$K(n+1,m) = K(n,m) + \sum_{r=1}^{m} K(n,r-1) = \sum_{r=0}^{m} K(n,r)$$

Now, using the induction hypothesis (IH) and the hockey-stick identity (HS), we have that

$$K(n+1,m) = \sum_{r=0}^{m} K(n,r) \overset{IH}{=} \sum_{r=0}^{m} \binom{n+r}{r}$$
$$\overset{HS}{=} \binom{n+m+1}{m} = \binom{(n+1)+m}{(n+1)}$$

which concludes the proof.
□

Using the Stirling's approximation of the factorial (SA) and assuming n and m grow at the same rate ($m \sim n$), we have the following asymptotic behavior for $K(n,m)$.

$$K(n,m) = \binom{n+m}{n} \overset{m \sim n}{\sim} \binom{2n}{n} = \frac{(2n)!}{(n!)^2}$$
$$\overset{SA}{\sim} \frac{\sqrt{2\pi(2n)}\left(\frac{2n}{e}\right)^{2n}}{2\pi n\left(\frac{n}{e}\right)^{2n}} = \frac{1}{\sqrt{\pi n}} 4^n$$

Supplementary benchmark results

Table 3 reports the performances for some general structure prediction algorithms on the training set from which we removed the non-pseudoknotted structures. The algorithms are ordered by F-score on this dataset which contains canonical and non-canonical base pairs.

Dataset composition

List of PDB identifiers in the training set:
`17ra 1a51 1a60 1a9l 1afx 1aju 1aqo 1ato 1atv 1atw 1bgz 1bn0 1bvj 1byj 1bz2 1c0o 1cq5 1d0t 1e4p 1e95 1ebq 1eht 1ehz 1ei2 1esh 1f1t 1f6x 1f84 1f85 1f9l 1fhk 1fmn 1hlx 1hs1 1hwq 1i3x 1idv 1ie1 1ik1 1ikd 1j4y 1jo7 1jox 1jtj 1jtw 1ju7 1jur 1k2g 1k4a 1k5i 1k6g 1k6h 1kaj 1kh6 1kks 1kos 1kp7 1kpy 1l1w 1l2x 1lc6 1ldz 1luu 1m5l 1m82 1me1 1mfj 1mfk 1mnx 1msy 1mt4 1n66 1n8x 1na2 1nbk 1oq0 1osw 1p5m 1p5n 1p5o 1pjy 1q75 1r2p 1r7w 1raw 1rfr 1rht 1rng 1roq 1s2f 1scl 1slo 1szy 1t28 1tbk 1tjz 1tlr 1txs 1u2a 1u9s 1uuu 1vop 1wks 1wts 1xhp 1xjr 1xwp 1xwu 1y27 1yg3 1ylg 1ymo 1ysv 1z2j 1z30 1z31 1z43 1zif 28sp 2a43 2au4 2b7g 2euy 2evy 2f87 2g1w 2gbh 2gis 2k4c 2l1v 2tpk 3d0u 3npn 3php 4e8q 4l81 4oji 4p5j 4qlm 4wfl 5l4o 5t83 5u3g 6dlq 6dme 6hbx 6n5n 6ol3 6tb7 7d81 7lva 7mlw 7uq6 8sp9`

List of PDB identifiers in the testing set:
`1esy 1qwa 1s9s 2aht 2fdt 2fey 2g1g 2gio 2gv3 2gvo 2hns 2hoj 2hua 2ixy 2ixz 2jr4 2jse 2jtp 2jwv 2jxv 2jym 2k5z 2k66 2kd8 2ke6 2khy 2kpc 2krl 2krp 2kry 2ktz 2kvn 2kx8 2kxm 2kzl 2l2j 2l3e 2l5z 2l6i 2lbj 2lbk 2lc8 2ldl 2ldt 2lhp 2li4 2ljj 2lk3 2lkr 2lp9 2lqz 2lu0 2lun 2lv0 2m12 2m21 2m22 2m24 2m4w 2m57 2m5u 2m8k 2meq 2mhi 2miy 2mn0 2mnc 2mqt 2mtj 2n1q 2n3r 2n4l 2n6s 2n6t 2n6w 2n6x 2n7m 2n8v 2nci 2o32 2o33 2oj7 2pcv 2qbz 2qh2 2qh4 2rlu 2ro2 2rpk 2rpt 2rrc 2rvo 2y95 2zy6 3e5c 3f2q 3gca 3sd3 3suh`

Supplementary table 3. Performances for all base pairs on single-stranded training dataset without non-pseudoknotted structures. Results are reported for all algorithms (top) and for mcdf when initialized with all algorithms (bottom). Mean scores are reported along with their standard deviation in a plus or minus fashion. The highest scores, lowest standard deviations and lowest computation times are in bold (ignoring Zipper). The last column reports the total computation time (top) and the difference between the global F-score measured when initiating mcdf with each algorithm versus the global F-score measured for each algorithm alone (bottom).

Initiation	Mean P	Mean R	Global P	Global R	Global F	Total time / F gain
RNAfold	0.829 ± 0.245	0.591 ± 0.184	0.837	**0.639**	**0.724**	**0.135**
Sfold	0.820 ± 0.297	0.557 ± 0.208	0.855	0.617	0.717	37.691
LinearFold	**0.893** ± 0.179	0.600 **± 0.129**	**0.858**	0.612	0.715	0.628
MXfold2	0.798 ± 0.295	0.564 ± 0.216	0.823	0.623	0.709	17.818
LinearPartition	0.875 ± 0.180	0.603 ± 0.132	0.834	0.589	0.690	0.938
CentroidFold	0.661 **± 0.166**	**0.624** ± 0.145	0.658	0.636	0.647	1.069
IPknot	0.768 ± 0.242	0.500 ± 0.220	0.754	0.373	0.499	38.725
MC-DuplexFold++	0.326 ± 0.237	0.366 ± 0.260	0.340	0.377	0.358	2.129
MC-FlashFold	0.315 ± 0.232	0.365 ± 0.259	0.324	0.380	0.350	0.341
Zipper	0.034 ± 0.080	0.039 ± 0.091	0.033	0.043	0.037	0.000
RNAfold	0.802 ± 0.254	0.593 ± 0.178	0.806	**0.652**	**0.721**	-0.003
Sfold	0.783 ± 0.308	0.561 ± 0.212	0.807	0.634	0.711	-0.006
LinearFold	**0.880** ± 0.180	0.599 **± 0.120**	**0.837**	0.612	0.707	-0.008
MXfold2	0.774 ± 0.298	0.568 ± 0.213	0.800	0.640	0.711	0.002
LinearPartition	0.865 ± 0.182	0.611 ± 0.124	0.816	0.612	0.700	0.010
CentroidFold	0.690 **± 0.165**	**0.616** ± 0.141	0.689	0.630	0.658	**0.011**
IPknot	0.760 ± 0.239	0.501 ± 0.221	0.748	0.374	0.499	0.000
MC-DuplexFold++	0.326 ± 0.236	0.362 ± 0.259	0.340	0.374	0.356	-0.002
MC-FlashFold	0.326 ± 0.237	0.366 ± 0.260	0.340	0.377	0.358	0.008
Zipper	0.143 ± 0.243	0.061 ± 0.102	0.116	0.070	0.088	0.051

3vrs 4jf2 4k27 4p9r 4pqv 4rzd 4xw7 5a17 5iem 5kmz 5kpy 5kqe 5lsn 5m0h 5n5c 5nz6 5uf3 5uzt 5v16 5wq1 6aas 6d3p 6fz0 6hag 6izp 6mxq 6pk9 6q8v 6u79 6ugi 6var 6vu1 6vvj 6w3m 6xwj 6xww 7d12 7dd4 7exy 7fhi 7k16 7k4l 7kub 7kuc 7kud 7lyj 7shx 7uga 7umc 7umd 7ume 7v06 7v9e 7wi9 8bwt 8clr 8cq1 8fcs 8hb8 8i43 8its 8scf 8sch

Training and testing datasets

Available at https://github.com/major-lab
